# Risk Factors and Prognostic Value of Swirl Sign in Traumatic Acute Epidural Hematoma

**DOI:** 10.3389/fneur.2020.543536

**Published:** 2020-11-09

**Authors:** Xuanzhi Wang, Ruixiang Ge, Jinlong Yuan, Shanshui Xu, Xinggen Fang, Yi Dai, Xiaochun Jiang

**Affiliations:** ^1^Department of Neurosurgery, The First Affiliated Hospital of USTC, Division of Life Sciences and Medicine, University of Science and Technology of China, Hefei, China; ^2^Department of Neurosurgery, The First Affiliated Hospital of Wannan Medical College (Yijishan Hospital of Wannan Medical College), Wuhu, China

**Keywords:** swirl sign, risk factor, prognostic, surgical, acute epidural hematoma

## Abstract

**Objective:** Acute epidural hematoma (AEDH) is one of the deadliest lesions in patients after traumatic brain injury. AEDH with swirl sign progresses rapidly and requires timely surgical treatment. This study aims to investigate the risk factors for the occurrence of AEDH with swirl sign and its prognostic value.

**Methods:** Retrospective analysis was performed on 131 AEDH patients, who were divided into swirl sign group and non-swirl sign group based on the brain computed tomographic (CT) scan. Patient information, including gender, age, hypertension, mechanism of injury, Glasgow Coma Scale (GCS) score on admission, time from injury to CT scan, pupillary light reactivity on admission, midline shift, location of hematoma, hematoma volume on admission, oral anticoagulation, and Glasgow Outcome Scale (GOS) score at 3 months were collected. Univariate analysis was used to determine the risk factors for the occurrence of swirl sign. The factors with *P* < 0.05 were recruited into the multivariate logistic regression analysis and predictive receiver operating characteristic (ROC) curve model.

**Results:** Univariate analysis demonstrated that the GCS score on admission (*P* = 0.007), pupillary light reactivity (*P* = 0.003), location of hematoma (*P* < 0.0001), and GOS score at 3 months (*P* = 0.007) were risk factors for the occurrence of swirl sign. Multivariate logistic regression model revealed that the location of hematoma (*OR* = 0.121; 95% CI: 0.019–0.786; *P* = 0.027) was an independent risk factor for swirl sign, and the occurrence of swirl sign was a significant predictor of unfavorable neurological outcomes (*OR* = 0.100; 95% CI: 0.016–0.630; *P* = 0.014). ROC curves demonstrated that the GCS score on admission (AUC = 0.655; 95% CI: 0.506–0.804), pupillary light reactivity (AUC = 0.625; 95% CI: 0.474–0.777) and location of hematoma (AUC = 0.788; 95% CI: 0.682–0.893) can predict the occurrence of swirl sign, respectively. Remarkably, the combination of these three factors (AUC = 0.829; 95% CI: 0.753–0.906) provided a greater power to predict the swirl sign.

**Conclusion:** GCS score on admission, pupillary light reactivity, and location of hematoma are risk factors for the occurrence of swirl sign, respectively. The combination of these three factors might be used to predict whether there is swirl sign in AEDH after traumatic brain injury. Furthermore, swirl sign can be used as an effective predictor of poor prognosis in patients.

## Introduction

Acute epidural hematoma (AEDH) is a common and severe complication of traumatic brain injury (TBI), occurring in 2.7–4% of all TBI patients (1). In the early days, mortality rate associated with AEDH was 86% (2), which has been reduced to 20% by modern surgical and anesthesia techniques, and timely surgical intervention (3, 4). The most common cause of artery bleeding in AEDH is middle meningeal injury. The rapid expansion of blood aggregated between the skull and dura mater causes a significant increase in intracranial pressure, leading to cerebral herniation, and even respiratory arrest. Fortunately, patients with AEDH who meet surgical indications and received timely surgical treatment have good prognosis. There is a special type of hematoma in AEDH named swirl sign, which is recognized as a hypodense area within a hyperdense hematoma on non-enhanced computed tomographic (CT) scan of the brain (5). The swirl sign of AEDH is easily identified on brain CT and its shape may be circular, linear or irregular (6). In recent years, there have been studies on swirl sign to predict the expansion of AEDH and to guide surgical treatment. Researchers have found that AEDH with swirl sign is an important predictor of hematoma enlargement and is of great significance in guiding emergency surgery (7). However, there are few studies on which factors are closely related to the occurrence of swirl sign and its guiding value for the prognosis of patients after head trauma. Therefore, the aim of this study is to present the risk factors and to analyze prognostic value of swirl sign in traumatic AEDH.

## Materials and Methods

### Patient Population

Due to the retrospective nature of the study, written informed consent was waived, the study protocol was approved by the Institutional Ethical Board of the Yijishan Hospital of Wannan Medical College. We retrospectively identified 131 patients with traumatic AEDH diagnosed by CT who underwent surgery or conservative treatment at the Department of Neurosurgery, Yijishan Hospital of Wannan Medical College (Wuhu, China) between January 2013 and December 2019. Of these, there were 17 AEDH patients with swirl sign. Data collected included gender, age, hypertension, mechanism of injury, Glasgow Coma Scale (GCS) score on admission, time from injury to CT scan, pupillary light reactivity on admission, midline shift, location of the hematoma, hematoma volume on admission, oral anticoagulation, and Glasgow Outcome Scale (GOS) score at 3 months. Exclusion criteria were as follows: (1) patients who suffered open craniocerebral injury; (2) subdural hematoma and/or intracerebral hematoma were found on admission CT scan; (3) existence of previous neurological diseases; (4) patients who did not receive neurosurgical treatment for AEDH; (5) had incomplete clinical data records or lost to follow up; and (6) multiple injuries (abbreviated injury score was ≥3).

### Treatment Procedure

All admitted patients underwent non-enhanced CT scans of brain and a set of neurological examinations, such as examination of limb muscle strength and muscle tone. Further medical treatments included intubation, mechanical ventilation and treatment of fluid resuscitation, as needed. Patients were then received surgery to evacuate the epidural hematoma or were admitted to the neurosurgical intensive care unit. The indications for surgery included: (i) Supratentorial hematoma volume ≥30 ml, (ii) midline shift >5 mm, (iii) abnormal pupillary reaction, (iv) compressed or absent basal cisterns, or (v) neurological deteriorations. Hematoma was removed by frontotemporal or temporal-parietal craniotomy. Intraoperative hemorrhage mainly originated from the main or branch of middle meningeal artery (MMA). Drainage tube was placed outside the dura mater if there was bleeding in the fracture suture of skull base. Necessity of bone flap removal was determined based on whether there was pre-operative cerebral herniation and the intra-operative intracranial pressure increased. The following GOS was used to evaluate outcome at 3 months after trauma: 1 death; 2 vegetative state; 3 severely disabled; 4 moderately disabled; and 5 good recovery (8). GOS 1–3 was defined as unfavorable outcome, whereas favorable outcome was defined as GOS score 4 and 5.

### Image Analysis

All patients underwent cranial CT scan to identify epidural hematoma. The volume of hematoma was calculated following the formula: (A × B × C / 2), in which A represents the maximum diameter of hematoma on axial CT, B represents the diameter perpendicular to A, and C represents the thickness of the hematoma (9). The midline shift was recorded by CT scan results, and the location of hematoma was confirmed by CT scan on admission. The swirl sign of AEDH was defined as a small hypoattenuating region with a swirl pattern within a large epidural hyperattenuating region. Neurosurgeons and neuroradiologists worked together to determine whether there was a swirl sign.

### Statistical Analyses

Data were analyzed using SPSS17.0 (SPSS Inc., Chicago, IL, USA) software. The Chi-square test was performed to analyze categorical variables. The *t*-test or Mann-Whitney *U*-test was used to analyze continuous variables. To identify the independent risk factors for swirl sign, factors with a *P* < 0.05 in univariate analysis were enrolled into the multivariate logistic regression analysis. A predictive receiver operating characteristic curve (ROC) model was established based on significant factors in univariate analysis. The discriminatory power of the model was evaluated by calculating the area under the curve (AUC), which was classified into 3 levels of predictive capability: excellent (AUC > 0.8), moderate (AUC: 0.7–0.8), and low (AUC: 0.6–0.7). Data were presented as odds ratio (OR) with 95% confidence interval (CI). SPSS17.0 software was used to analyze the ROC curves. *P* < 0.05 was considered to indicate statistical significance.

## Results

**Figure 1:**
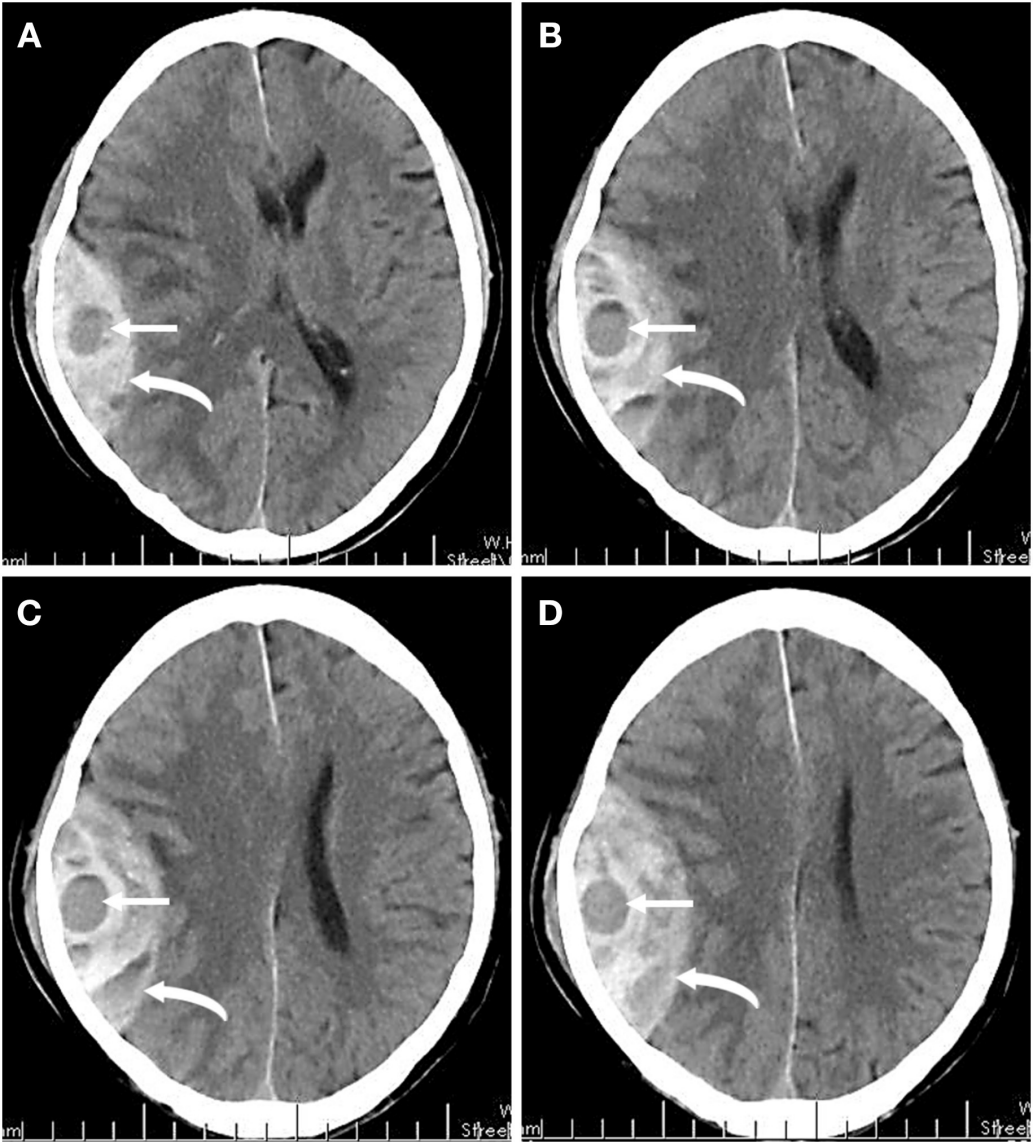
**(A–D)** A series of non-enhanced brain CT images of one patient at 2 h after head trauma. Right temporoparietal hyperattenuating epidural hematoma (curved arrows) with local hypoattenuating (straight arrows) represents the swirl sign.

A total of 131 patients with AEDH met the inclusion criteria. The general demographic, clinical, and radiological data of the patients are shown in Table 1. Among them, 81 (61.83%) patients were male and 50 (38.17%) were female, with a mean age of 42.15 ± 9.78 years old. Of the 131 patients, 83 (63.36%) suffered traffic accidents and 48 (36.64%) fell from height. Hypertension was presented in 42 (32.06%) of all patients. It is noteworthy that there were 17 (12.98%) cases showed swirl sign on non-enhanced brain CT scans (Figure 1). Of the 17 patients with swirl sign, only 3 (17.65%) patients had a history of oral anticoagulation.

**Table 1:** Clinical and radiological characteristics of patients with swirl sign and without swirl sign [*n* (%)].

**Variables**	**Swirl sign (*n* = 17)**	**Non-swirl sign (*n* = 114)**	**t/χ^2^**	***P*-value**
Age, mean ± SD	41.52 ± 9.34	42.76 ± 10.37	0.465	0.642
**Gender**, ***n*** **(%)**				
Male	12 (70.59)	69 (60.53)	0.635	0.426
Female	5 (29.41)	45 (39.47)		
**Hypertension**, ***n*** **(%)**				
Yes	4 (23.53)	38 (33.33)	0.653	0.419
No	13 (76.47)	76 (66.67)		
**Mechanism of injury**, ***n*** **(%)**				
Traffic accident	10 (58.82)	73 (64.04)	0.173	0.677
Fall	7 (41.18)	41 (35.96)		
**Time from injury to CT scan**, ***n*** **(%)**				
<3 h	13 (76.47)	61 (53.51)	3.173	0.075
≥3 h	4 (23.53)	53 (46.49)		
**Oral anticoagulation**, ***n*** **(%)**				
Yes	3 (17.65)	16 (14.04)	0.156	0.693
No	14 (82.35)	98 (85.96)		
GCS scores on admission, mean ± SD	8.53 ± 0.52	10.15 ± 0.19	3.11	0.002
**Pupillary light reactivity**, ***n*** **(%)**				
None	1 (5.88)	3 (2.63)	11.790	0.003
Unilateral	8 (47.06)	16 (14.04)		
Bilateral	8 (47.06)	95 (83.33)		
**Midline shift**, ***n*** **(%)**				
<5 mm	10 (58.82)	74 (64.91)	0.238	0.625
≥5 mm	7 (41.18)	40 (35.09)		
**Location of hematoma**, ***n*** **(%)**				
Temporal/temporoparietal	14 (82.35)	31 (27.19)	19.96	<0.0001
Frontal/parietal/occipital	3 (17.65)	83 (72.81)		
Hematoma volume (ml), mean ± SD	40.24 ± 7.59	36.63 ± 11.13	1.287	0.200
**GOS score at 3 months**, ***n*** **(%)**				
Favorable	12 (70.59)	105 (92.11)	7.176	0.007
Unfavorable	5 (29.41)	9 (7.89)		

*CT, computed tomography; GCS, Glasgow Coma Scale; GOS, Glasgow Outcome Scale*.

The univariate analysis of clinical and radiological factors demonstrated that the swirl sign was significantly associated with the GCS score on admission (*P* = 0.007), pupillary light reactivity (*P* = 0.003), location of hematoma (*P* < 0.0001), and GOS score at 3 months (*P* = 0.007). There was no significant correlation between the occurrence of swirl sign and age (*P* = 0.642), gender (*P* = 0.426), hypertension (*P* = 0.419), mechanism of injury (*P* = 0.677), time from injury to CT scan (*P* = 0.075), oral anticoagulation (*P* = 0.693), midline shift (*P* = 0.625), or hematoma volume (*P* = 0.200). Detailed analyses are as follows: of the 17 patients with swirl sign, 9 (52.94%) had an admission GCS score of 3–8, while only 25 (21.93%) of 114 patients without swirl sign had a GCS score on admission of 3–8. For admission pupillary light reactivity, there were 83.33% (95 of 114) of patients in non-swirl sign group had bilateral pupil reactivity, while this number was 47.06% (8 of 17) for patients in swirl sign group. Moreover, 88.24% (14 of 17) of patients with swirl sign had temporal or temporoparietal hematoma, which was significantly higher than 27.19% (31 of 114) of patients without swirl sign. GOS score was evaluated at 3 months after injury, unfavorable neurological outcome was observed in 29.41% (5 of 17) of patients with swirl sign and in 7.89% (9 of 114) of those without swirl sign. Additionally, the positive and negative predictive values of swirl sign in predicting poor outcomes were 35.71% (5 of 14) and 89.74% (105 of 117), respectively. Furthermore, the multivariate logistic regression analysis revealed that the location of hematoma (*OR* = 0.121; 95% CI: 0.019–0.786; *P* = 0.027) was an independent risk factor for swirl sign, and GOS score at 3 months (*OR* = 0.100; 95% CI: 0.016–0.630; *P* = 0.014) was independently associated with swirl sign (Table 2). These results indicated that the occurrence of swirl sign was a significant predictor of unfavorable neurological outcome.

**Table 2:** Multivariate logistic regression model for factors associated with swirl sign.

**Factors**	**OR**	**95% CI**	***P*-value**
GCS score on admission	0.300	0.048–1.888	0.200
Pupillary light reactivity	0.299	0.077–1.162	0.081
Location of hematoma	0.121	0.019–0.786	0.027
GOS score at 3 months	0.100	0.016–0.630	0.014

*GCS, Glasgow Coma Scale; GOS, Glasgow Outcome Scale; OR, odds ratio; CI, confidence interval*.

**Figure 2:**
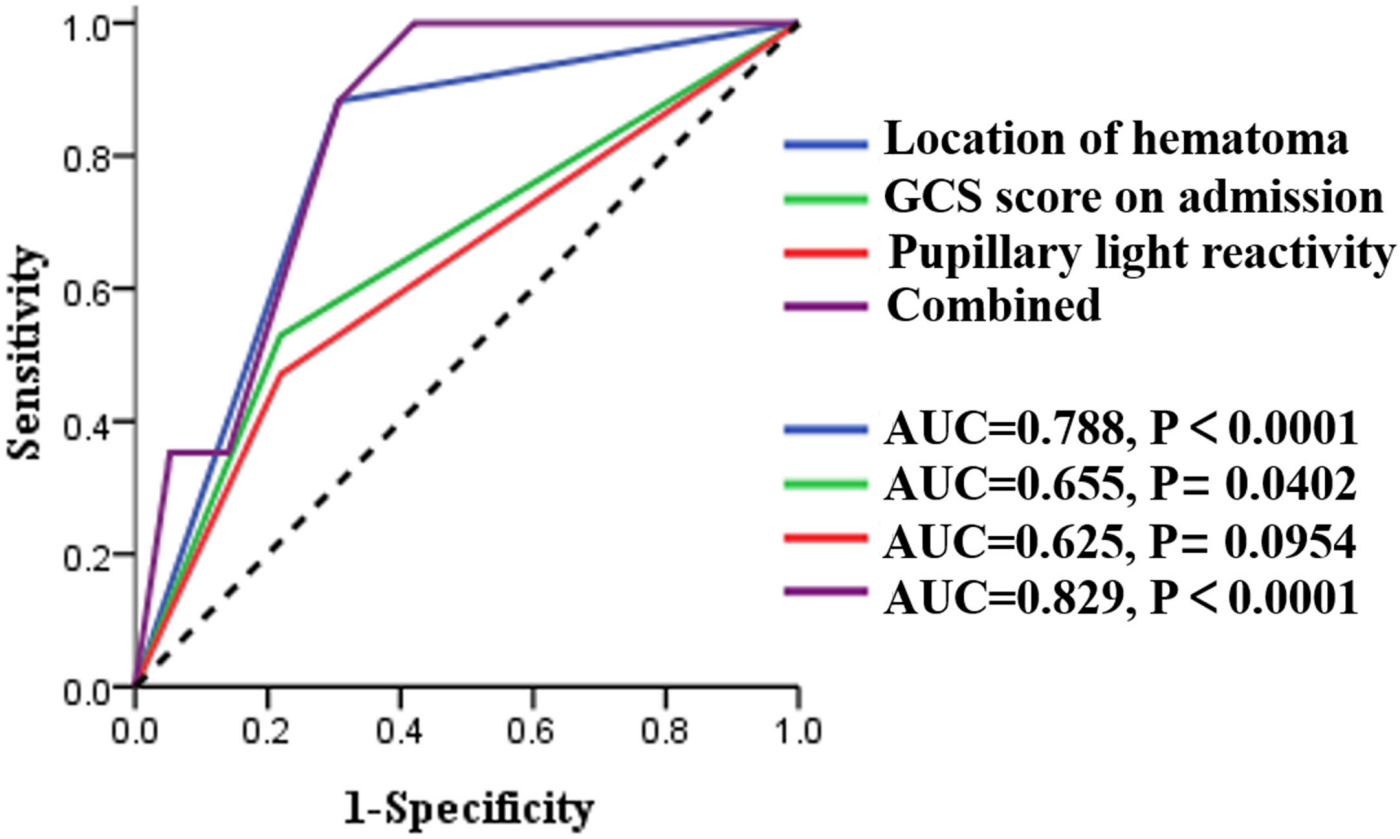
ROC curve models of the three risk factors acquired from univariate analysis. AUC, area under the curve; CI, confidence interval; GCS, Glasgow Coma Scale.

To further investigate the value of related risk factors in predicting the occurrence of swirl sign, ROC curve models were created and the AUC values were calculated (Figure 2). The AUC values were 0.788 for location of hematoma (95% CI: 0.682–0.893), 0.655 for GCS score on admission (95% CI: 0.506–0.804), and 0.625 for pupillary light reactivity (95% CI: 0.474–0.777), respectively (Table 3). The combined AUC value was 0.829 (95% CI: 0.753–0.906), with a sensitivity of 97.83%, and specificity of 57.91%. These results showed that the combination of these three factors has good predictive power for swirl sign.

**Table 3:** The AUC values under the ROC curves.

**Factors**	**AUC**	**SE**	**95% CI**
GCS scores on admission	0.655	0.076	0.506–0.804
Pupillary light reactivity	0.625	0.077	0.474–0.777
Location of hematoma	0.788	0.054	0.682–0.893
Combined	0.829	0.039	0.753–0.906

*AUC, area under the curve; CI, confidence interval; GCS, Glasgow Coma Scale; SE, standard error; ROC, operating characteristic curve*.

## Discussion

Traumatic AEDH is common in clinic, and aggressive treatment after confirmed diagnosis can improve the survival rate of patients (8). The brain CT scan of patients on admission showed the presence of a hypodense area within the hyperdense epidural hematoma (swirl sign), which indicated that there was active bleeding in the hematoma (9). If the swirl sign is not detected in time in the process of observation and treatment, it will result in hematoma expansion and brain hernia, leading to disability or even death of patients. In our study, 29.41% of patients with swirl sign had an unfavorable neurological outcome, which was significantly higher than 7.89% of patients without swirl sign. This demonstrated that the occurrence of swirl sign was significantly associated with high morbidity and mortality rates and it could be used as a potential predictor of unfavorable outcomes in patients (10). Therefore, it is important to investigate the risk factors of swirl sign for guiding surgical management of patients.

AEDH patients with swirl sign have rapid disease progression, often shown as consciousness disorder in a short time after injury, and there was no classic “lucid interval” of epidural hematoma (11). Ndoumbe et al. retrospectively analyzed 46 patients with traumatic AEDH based on GCS score. The results demonstrated that the GCS score was a good predictor of prognosis, and all survivors with unfavorable outcome had GCS ≤ 8 on admission (12). Our results showed that 52.94% of patients with swirl sign had a GSC score of 3–8 on admission, a percentage significantly higher than that of patients without swirl sign (21.93%). This suggested that compared with patients without swirl sign, patients with swirl sign were in more serious conditions and had more severe comatose on admission. Further statistical analysis showed that GCS score was a risk factor for the occurrence of swirl sign, but not an independent risk factor. The pupillary light reactivity after head trauma plays an important role in the occurrence and localization of intracranial hematoma (13). Monitoring of pupillary light reactivity provides information about secondary insults (such as high intracranial pressure), and sustained or newfound pupillary mydriasis is a validated predictor of worse functional outcome (14). Rosyidi's group retrospectively analyzed 268 patients with AEDH diagnosed by CT scan and found that 29% of patients had anisocoria during hospitalization (15). In our study, 16.67% of patients without swirl sign had abnormal pupillary light reactivity on admission, while 52.94% of patients with swirl sign had pupillary mydriasis on admission. This indicated that compared with AEDH without swirl sign, AEDH with swirl sign was more likely to result in a sharp increase in intracranial pressure and abnormal pupillary light reactivity. Similar to GCS score on admission, multivariate regression analysis suggested that pupillary light reactivity was a risk factor for the occurrence of swirl sign, but not an independent risk factor. Most AEDH is caused by tearing of the main or branch of the MMA (16). Since the MMA mainly travels under the temporal bone and parietal bone, temporal, or temporoparietal AEDH is most often seen. Rosyidi et al. investigated 268 patients with AEDH and found that 38.05% of epidural hematoma (EDH) occurred in the temporal or temporoparietal location (15). Our results showed that in all patients with swirl sign, 82.35% of EDH was temporal or temporoparietal, which was significantly higher than 27.19%, that of patients without swirl sign. Interestingly, of the 131 patients we studied, 34.35% (45 of 131) of EDH was temporal or temporoparietal, which was consistent with results from previous studies (15, 17). These studies indicated that the swirl sign was significantly associated with the location of hematoma. Additionally, further analysis showed that the location of hematoma was an independent risk factor for the occurrence of swirl sign.

Finally, a ROC model was established in this study to further evaluate the accuracy of these factors in predicting the occurrence of swirl sign. According to the AUC value, GCS score on admission and pupillary light reactivity had a low predictive accuracy for swirl sign, whereas the location of hematoma had a moderate predictive value. Notably, the combination of the three risk factors had excellent accuracy in predicting the occurrence of swirl sign.

There are differences in clinical characteristics and radiological manifestations between AEDH with and without swirl sign, which should be highly valued by clinicians. The time of surgery for AEDH with swirl sign should be more flexible, and should not be restricted to the surgical indications specified in the guidelines (7). Once identified, surgery should be performed immediately to remove hematoma and stop bleeding. For this type of surgery, the design of the preoperative bone flap should include the location of swirl sign, which indicates the source of bleeding and should be fully exposed to facilitate complete hemostasis under direct vision during the operation.

For AEDH with swirl sign, although it is treated by neurosurgeons, it is often identified first by radiologists during clinical check-up. Therefore, timely communication between radiologists and neurosurgeons is needed to shorten the rescue time and improve the prognosis of patients.

There are several limitations in our study. Firstly, this is a retrospective study that may introduce bias in patient selection and data collection. For instance, different neurosurgeons might give different assessment for the observational indicators (e.g., GCS scores and pupillary light reactivity, etc.). Secondly, our data came from a single center, and the lost of some data during patient follow-up resulted in a reduction in the number of patients included in this study, which limited the power to identify potential risk factors for swirl sign. Therefore, our results need to be further verified by prospective, large-sample, and multicenter studies.

## Conclusion

The present study demonstrates that the GCS score on admission, pupillary light reactivity and location of hematoma are all risk factors for the occurrence of swirl sign, respectively. Specifically, the location of hematoma is an independent risk factor for swirl sign, which can be used as an effective predictor of poor prognosis in patients. GCS score on admission, pupillary light reactivity and location of hematoma can predict the occurrence of swirl sign, respectively. Importantly, the combination of the three factors provides a greater power to predict the swirl sign. In general, sufficient attention should be paid by the neurosurgeons to AEDH with swirl sign.

## Data Availability Statement

All datasets generated for this study are included in the article/supplementary material.

## Ethics Statement

Due to the retrospective nature of the study, written informed consent was waived, the study protocol was approved by the Institutional Ethical Board of the Yijishan Hospital of Wannan Medical College.

## Author Contributions

XW contributed to study design, data collection, imaging analysis, statistical analysis, and manuscript drafting. RG and JY contributed to data collection and statistical analysis. SX, XF, YD, and XJ took part in conception and design, assisted with image analyses, and manuscript revision. All authors read and approved the final manuscript.

## Conflict of Interest

The authors declare that the research was conducted in the absence of any commercial or financial relationships that could be construed as a potential conflict of interest.
